# Prognostic factors for survival after allogeneic transplantation in acute lymphoblastic leukemia

**DOI:** 10.1038/s41409-020-01101-z

**Published:** 2020-10-31

**Authors:** C. Greil, M. Engelhardt, G. Ihorst, J. Duque-Afonso, K. Shoumariyeh, H. Bertz, R. Marks, R. Zeiser, J. Duyster, J. Finke, R. Wäsch

**Affiliations:** 1 Department of Hematology, Oncology and Stem Cell Transplantation, Medical Center, University of Freiburg, Freiburg, Germany; 2 Faculty of Medicine, University of Freiburg, Freiburg, Germany; 3 Clinical Trials Unit, Faculty of Medicine, University of Freiburg, Freiburg, Germany

**Keywords:** Acute lymphocytic leukaemia, Risk factors

## Abstract

Allogeneic stem cell transplantation (allo-SCT) offers a curative option in adult patients with acute lymphoblastic leukemia (ALL). Prognostic factors for survival after allo-SCT have not been sufficiently defined: pheno-/genotype, patients´ age, conditioning regimens and remission at allo-SCT are under discussion. We analyzed the outcome of 180 consecutive adult ALL-patients undergoing allo-SCT at our center between 1995 and 2018 to identify specific prognostic factors. In our cohort 19% were older than 55 years, 28% had Philadelphia-positive B-ALL, 24% T-ALL. 54% were transplanted in first complete remission (CR1), 13% in CR2 after salvage therapy, 31% reached no remission (8% within first-line, 23% within salvage therapy). In 66% conditioning contained total body irradiation (TBI). With a median follow-up of 10 years, we observed an overall survival of 33% at 10 years, and a progression free survival of 31%. The cumulative incidence of relapse was 41% at 10 years, the cumulative incidence of non-relapse mortality 28%. Acute graft-versus-host disease (GvHD) II°–IV° occurred in 31%, moderate/severe chronic GvHD in 27%. Survival was better in patients reaching CR before allo-SCT and in those receiving TBI. No difference between patients younger/older than 55 years and between different phenotypes was observed. Survival after allo-SCT improved considerably over the last decades.

## Introduction

Acute lymphoblastic leukemia (ALL) is a rare hematological disorder most commonly diagnosed in children [1, 2]. It is a heterogeneous disease, distinct biologic subtypes are characterized by different immunophenotypic and genetic features requiring specific treatment approaches [1, 2]. Unfavorable genetic aberrations are more frequent in adult ALL as compared to childhood disease [3], leading to a less favorable outcome. In addition, intensive chemotherapy regimens adapted from pediatric protocols, especially required in those high-risk constellations, maybe not applicable to elderly patients due to concomitant diseases and organ dysfunction. Thus, long-term survival in adults is still poor with a 5-year (y) overall survival (OS) of 30–40%, although response rates considerably improved over the last decades with the development of risk-adapted treatment strategies [4].

In the last years, the therapeutic landscape in ALL considerably changed especially in the relapsed/refractory situation with the introduction of novel innovative therapeutic options like antibodies [5, 6] or CAR T-cells [7]. Yet, CAR T-cells are still not approved in ALL-patients older than 25 years due to a high toxicity. Thus, allogeneic stem cell transplantation (allo-SCT) remains an important potentially curative immunotherapeutic approach: it allows a myeloablative therapy contributing to disease eradication and donor lymphocytes may sustain a graft-versus-leukemia (GvL)-effect [8]. However, survival after allo-SCT is impaired due to treatment-associated toxicity, in particular graft-versus-host disease (GvHD) and infections, resulting in a non-relapse mortality (NRM) of ~35% in adults [9]. Survival of ALL-patients generally improved over time [10, 11] as a result of an optimized risk stratification and patient selection, adapted treatment protocols, growing availability of optimal donors, reduced treatment-associated toxicity, intensified supportive care and adjusted co-medication. For patients who reached a complete remission after first induction (CR1) or after salvage therapy in case of relapse (CR2), the 5y-OS is ~45% [12].

Allo-SCT and conventional post-remission chemotherapy in adult ALL-patients have not been directly compared in randomized trials. Instead, patients were assigned to the appropriate treatment based on the presence of a human leukocyte antigen (HLA)-matched sibling donor. Characteristics associated with a survival advantage for allo-SCT as compared to a less intensive consolidation were identified in retrospective subgroup analyses [9, 13, 14]. Putative factors defining those patients who are at high risk of relapse after conventional treatment are high leukocyte counts, involvement of the central nervous system, diagnosis of pro-B-, early or mature T-ALL, late achievement of CR1, detection of t(9;22) or t(4;11), a *BCR-ABL*1-like gene signature, hypodiploidy and the presence of minimal residual disease (MRD) [15–21]. Thus, allo-SCT is recommended as consolidation therapy in CR1 for those patients, in particular in Philadelphia chromosome (Ph)-positive ALL which used to be defined as a very high-risk disease, but has an improved outcome since the introduction of tyrosine kinase inhibitors (TKI) [9, 22–24]. Patients without evidence of high-risk features may still relapse and then have a very poor survival [25]. This can be significantly improved with allo-SCT in CR2 as compared to conventional chemotherapy [15, 26].

Additional disease- and patient-related prognostic factors to predict and optimize survival after allo-SCT need to be defined. To address this need, we here analyzed the outcome of the 180 adult ALL-patients who underwent allo-SCT at our center in the last 20 years with regard to treatment response, survival, and toxicity. We performed subset analyses to identify patients that benefited most from that therapy.

## Methods

### Patients’ description and data source

We retrospectively analyzed 180 consecutive adult ALL-patients who received allo-SCT between 1995 and 2018 at the University Hospital of Freiburg. Data were analyzed as of January 2019. The analysis was carried out according to the Declaration of Helsinki and Good Clinical Practice guidelines. Patient data were retrieved from our institution’s electronic medical records. Patients received follow-up on a regular basis. All patients gave their written informed consent for institutional-initiated research studies, approved by our institutional review board. Twenty-three percent were treated within prospective GMALL (German ALL study group)-trials, 11% were included in the GMALL-registry (Supplementary Table 1). The proportion of study participants in our total allo-cohort was rather low as treatment was often initiated in smaller hospitals and patients were only referred to our university center in case of high-risk or relapsed/refractory disease. Outside of trials, treatment was performed in line with the national therapeutic guidelines according to the GMALL-recommendations. MRD was assessed in all patients: In 47% Ig-/TR-gene rearrangements were analyzed via RQ-PCR in the GMALL-reference lab with a threshold of <10^−4^ [27]. In 53% surface marker analyses by flow cytometry were performed in our local lab and a detection of <0.1% of the initial aberrant phenotype was defined as MRD-negative (Table 1). All samples were gated on CD19 and a four-color panel was applied [28].

**Table 1:** Patients´ characteristics and outcome (*n* = 180).

**Sex** (%)	Male/Female	109/71 (61/39)
**Median age** (range)	[Years]	37 (16–76)
**Phenotype** (%)	B-ALL Ph neg	86 (48)
	B-ALL Ph pos	50 (28)
	T-ALL	44 (24)
**Remission state at allo-SCT** (%)	CR1 MRD neg PCR^a^/FACS^b^ CR1 MRD pos^a+b^ Primary refractory	39/30 (22/16) 33 (18) 14 (8)
	CR2 MRD neg PCR^a^/FACS^b^ CR2 MRD pos^a+b^ Relapsed refractory	4/14 (2/8) 5 (3) 41 (23)
**Donor** (%)	Related HLA-identical/nonidentical Unrelated HLA-identical/nonidentical	54/5 (30/3) 86/35 (48/19)
**Stem cell source** (%)	PB/BM	153/27 (85/15)
**TBI-based conditioning** (%)	Yes/no	119/61 (66/34)
**GvHD prophylaxis** (%)	CyA + ATG + Alemtuzumab	178 (99) 80 (44) 47 (26)
**Alive**/**dead** (%)		63/117 (35/65)
**Primary cause of death** (%)	**PD**	62 (34)
	**NRM**	55 (31)
**Death within 100 days after allo-SCT** (%)		29 (16)
**GvHD** (%)	**Acute** ≤ I°/≥ II°	124/56 (69/31)
	**Chronic** no or mild/moderate or severe	131/49 (73/27)

*ALL* acute lymphoblastic leukemia, *Ph* Philadelphia chromosome, *pos* positive, *neg* negative, *allo-SCT* allogeneic stem cell transplantation, *CR* complete remission, *CR1* after first induction therapy, *CR2* after salvage therapy in case of relapse, *MRD* minimal residual disease, *HLA* human leukocyte antigen, *PB* peripheral blood, *BM* bone marrow, *TBI* total body irradiation, *GvHD* graft-versus-host disease, *CyA* cyclosporin A, *ATG* Anti-thymocyte Globulin, *PD* progressive disease, *NRM* non-relapse-related mortality.

^a^Analysis of Ig-/TR-gene rearrangements via RQ-PCR in 85/180 patients = 47%.

^b^Analysis via surface marker analysis by flow cytometry in 95/180 patients = 53%.

### Statistical analysis

Data were analyzed using SAS statistical software version 9.4 (SAS Institute Inc., Cary, NC, USA). OS and progression-free survival (PFS) were calculated as time from allo-SCT to death from any cause and first observation of relapse or death. NRM was defined as death without progressive disease. Patients without observation of the event of interest at the last follow-up were treated as censored observations. OS and PFS rates were estimated and reported using the Kaplan-Meier method. Relapse and NRM were considered to be competing risks, rates were estimated as cumulative incidence rates using Aalen Johansen estimator and compared with Fine and Gray regression models for competing risks. Prognostic factors were investigated in multivariate regression models adjusting for the timepoint of allo-SCT. *P* values of <0.05 were considered as statistically significant.

## Results

### Patients´ characteristics

The median age of our cohort was 37 years, 19% were older than 55 years. We observed a male predominance (61% vs. 39%; Table 1). In 64%, allo-SCT was planned within the first-line therapy. With evidence of t(9;22), 28% were classified as very high-risk. Twenty-six percent were defined as high-risk according to the phenotype (proB-, early, mature T-ALL; Supplementary Table 1). Therefore, allo-SCT was mostly performed shortly after initial diagnosis (median 6.2 months; Supplementary Table 1).

### Previous treatment and transplantation procedure

The majority of patients were treated with cyclophosphamide-, daunorubicin-, vincristine-, asparaginase-, and cytarabine-containing induction protocols according to the GMALL-protocols. Fifty-eight percent of the Ph-positive cases received TKI-containing protocols before allo-SCT, mostly imatinib (Supplementary Table 1), 42% were treated in the pre-TKI era. In 66% of the patients, chemotherapy before allo-SCT was combined with rituximab (Supplementary Table 1). In 48%, whole brain irradiation (WBI) was performed. In many cases induction therapy was conducted without WBI when a total body irradiation (TBI)-based conditioning therapy was planned upfront due to a high-risk constellation. Only 4% received prior irradiation of a mediastinal tumor (Supplementary Table 1). A detailed description of salvage therapies in case of relapse is listed in Supplementary Table 1.

Approximately two-thirds of the total cohort reached CR prior to allo-SCT (56% CR1; 13% CR2): 48% were determined as MRD-negative (38% CR1; 10% CR2), in 24% verified via RQ-PCR and in 24% via surface marker analysis by flow cytometry (Table 1). The remaining third was admitted to allo-SCT with relapsed/refractory disease (Table 1).

For the majority of patients, donor stem cells were harvested from the peripheral blood, only 15% received bone marrow (BM), mainly in the earlier years (85% before 2000). Thirty percent of all transplantations were conducted with HLA-matched related donors, 3% with haploidentical related donors, 48% with HLA-matched, and 19% with HLA-mismatched unrelated donors (Table 1). All patients received a myeloablative conditioning, in 3% a reduced-toxicity myeloablative conditioning was chosen due to age and comorbidities. In 66%, conditioning was TBI-based with a dose of 12 Gray, only one patient received 8 Gray (Table 1). Details on conditioning chemotherapeutics are depicted in Supplementary Table 1.

Cyclosporin A was used for GvHD-prophylaxis, either in combination with alemtuzumab or methotrexate or mycophenolate mofetil with or without antithymocyte globulin (Table 1). Further transplant data such as donor sex, CMV-status, and hematological recovery are summarized in Supplementary Table 1.

### Treatment response, survival and treatment-related toxicity

**Fig. 1:**
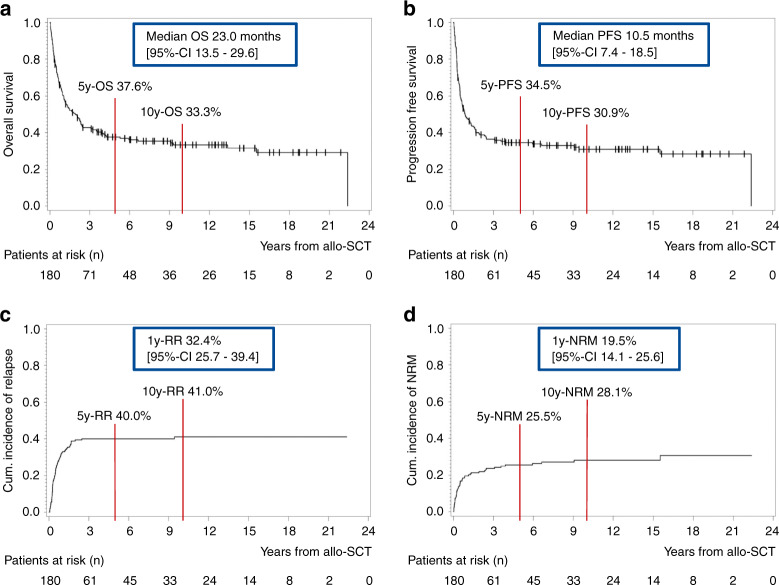
Outcome analysis of the entire cohort. **a** Kaplan–Meier estimates for OS, **b** Kaplan–Meier estimates for PFS, **c** cumulative incidence of relapse, **d** cumulative incidence of NRM. *OS* overall survival, *PFS* progression free survival, *RR* relapse rate, *NRM* non-relapse-related mortality, *y* year, *CI* confidence interval.

At the time of analysis, 35% of all patients were still alive. In 34%, the primary cause of death was relapsed/refractory disease. The NRM was 31% (Table 1). Best response determined at median one month after allo-SCT (range 0.7–13 months) was CR in 86% of the patients, in 78% with MRD-negativity. With a median follow-up of 10.2 years [95%-confidence interval (CI) 7.9–12.7 years], the median OS was 23 months [95%-CI 13.5–29.6 months] and PFS 10.5 months [95%-CI 7.4–18.5 months] (Fig. 1). In both survival curves, a plateau was observed, indicating a long-term survival in approximately one-third of the cohort, with an OS of 33.3% and PFS of 30.9% at 10 years (Fig. 1). Accordingly, the cumulative incidence of relapse after 1 year was 32.4% [95%-CI 25.7–39.4%], which reached a plateau after 5 years at 40.0% (Fig. 1). The cumulative incidence of NRM showed a similar trend with 19.5% after 1 year [95%-CI 14.1–25.6%] and 25.5% after 5 years (Fig. 1). Acute GvHD (aGvHD) was documented in 52% of the patients, mostly affecting the skin or gut. The majority had mild symptoms, aGvHD ≥II° occurred in 31% (Table 1). Forty percent of the patients showed signs of chronic GvHD (cGvHD), in 27% with moderate/severe course (Table 1).

### Analysis of putative prognostic factors and comparison of different therapies

Due to the long observation period, our cohort consisted of a heterogenous group with various disease- and patient-related risk factors, treated according to evolving therapeutic guidelines. Therefore, various subset analyses were performed to differentiate between those properties, to define different risk groups and to identify patients that might benefit most from allo-SCT.

### Therapy line and remission status prior to allo-SCT

First, we compared the outcome of patients transplanted upfront due to a high-risk constellation with those who received allo-SCT after salvage therapy for relapsed/refractory disease (Supplementary Fig. 1). We observed a significantly better survival for patients who underwent allo-SCT as part of the first-line therapy as compared to relapsed patients (*p* < 0.0001; Supplementary Fig. 1A, B) due to a significantly lower cumulative incidence of relapse (*p* < 0.0001; Supplementary Fig. 1C). The cumulative incidence of NRM did not differ between the two groups (Supplementary Fig. 1D).

**Fig. 2:**
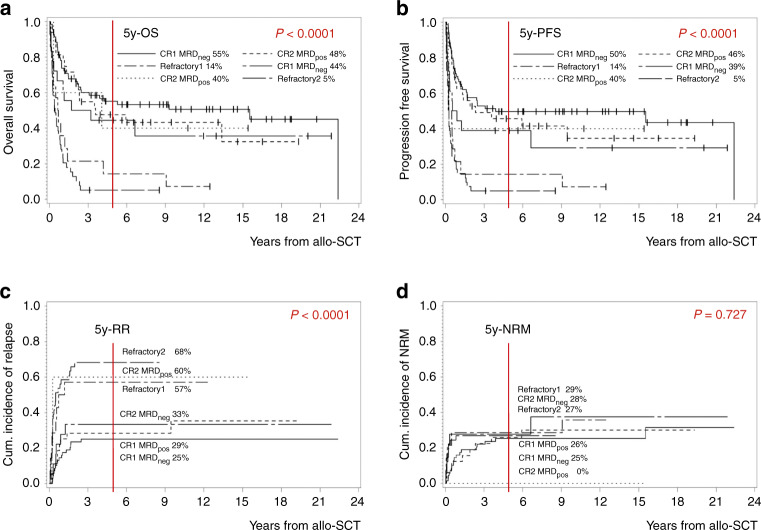
Impact of remission status at the timepoint of allo-SCT and therapy line on outcome: patients who reached MRD-negative^1^ CR1 (*n* = 69) vs. those with MRD-positive CR1 (*n* = 33) vs. those with primary refractory disease (*n* = 14) vs. patients transplanted in case of relapse who reached MRD-negative^1^ CR2 (*n* = 18) vs. those with MRD-positive CR2 (*n* = 5) vs. those with relapsed refractory disease (*n* = 41). **a** Kaplan–Meier estimates for OS, **b** Kaplan–Meier estimates for PFS, **c** cumulative incidence of relapse, **d** cumulative incidence of NRM. *Allo-SCT* allogeneic transplantation, *OS* overall survival, *PFS* progression free survival, *RR* relapse rate, *NRM* non-relapse-related mortality; *y*year, *CR* complete remission, *MRD* minimal residual disease, *neg* negative, *pos* positive. ^1^Analysis of Ig-/TR-gene rearrangements via RQ-PCR and via surface marker analysis by flow cytometry.

In addition, the influence of response to induction therapy prior to allo-SCT on survival was assessed. We first differentiated between patients who reached a molecular or hematological CR vs. those who did not achieve a remission: OS and PFS were impaired in patients with induction failure or relapse as compared to those who achieved CR, again due to a higher cumulative incidence of relapse. Taking both therapy line and remission status into account, OS was similar in patients reaching CR prior to allo-SCT irrespective whether it was performed during first-line therapy or after salvage therapy for relapsed/refractory disease and inferior in patients who did not achieve a remission (5y-OS in MRD-negative CR1 55.1%, MRD-positive CR1 47.5%, MRD-negative CR2 44.4%, MRD-positive CR2 40.0% vs. no remission in first-line therapy 14.3% or in salvage situation 5.1%, *p* < 0.0001; Fig. 2a). The same was true for PFS (5y-PFS in MRD-negative CR1 49.6%, MRD-positive CR1 45.6%, MRD-negative CR2 38.9%, MRD-positive CR2 40.0% vs. no remission in first-line therapy 14.3% or in salvage situation 4.9%, *p* < 0.0001; Fig. 2b). The cumulative incidence of relapse was significantly higher in patients transplanted with active disease as compared to those who previously achieved a remission (5y-relapse rate (RR) in MRD-negative CR1 25.1%, MRD-positive CR1 28.5%, MRD-negative CR2 33.3%, MRD-positive CR2 60.0% vs. no remission in first-line therapy 60.0% or in salvage situation 68.3%, *p* < 0.0001; Fig. 2c). The cumulative incidence of NRM was similar among all groups (5y-NRM in MRD-negative CR1 25.3%, MRD-positive CR1 25.9%, MRD-negative CR2 27.8%, MRD-positive CR2 0.0% vs. no remission in first-line therapy 28.6% or in salvage situation 26.8%, *p* = 0.727; Fig. 2d). The deviating results for relapse and NRM in the subgroup of MRD-positive CR2 are likely explained by the limited group number of only five patients.

To further investigate the impact of MRD, we analyzed the 62 patients who reached CR1 with available MRD-assessment via RQ-PCR separately (excluding assessment by flow cytometry). Here, we observed a small increase in survival differences for the MRD-negative cohort (5y-OS in MRD-negative CR1 60.6% vs. 50.8% in MRD-positive CR1, *p* = 0.551; 5y-PFS in MRD-negative CR1 55.2% vs. 48.4% in MRD-positive CR1, *p* = 0.485), although differences were still statistically insignificant.

### Pre-transplant therapy

As expected, in the 50 Ph-positive patients, OS and PFS were significantly improved by addition of a TKI (both *p* < 0.05; Supplementary Fig. 2A, B). Similarly, the cumulative incidence of relapse decreased (*p* < 0.05; Supplementary Fig. 2C), while the toxicity remained comparable (Supplementary Fig. 2D).

### Conditioning regimen

**Fig. 3:**
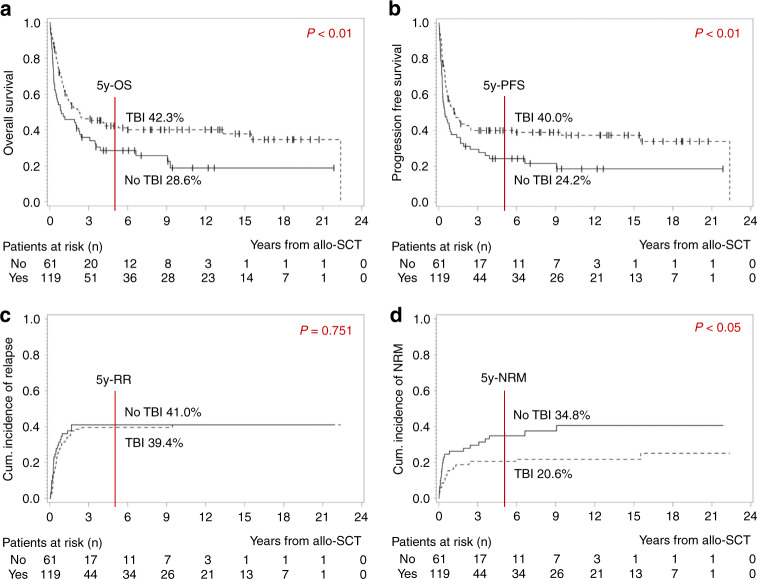
Impact of conditioning therapy on outcome: Patients who received TBI-based conditioning (*n* = 119) vs. those without TBI (*n* = 61). **a** Kaplan–Meier estimates for OS, **b** Kaplan–Meier estimates for PFS, **c** cumulative incidence of relapse, **d** cumulative incidence of NRM. *OS* overall survival, *PFS* progression free survival, *RR* relapse rate, *NRM* non-relapse-related mortality, *y* year, *TBI* total body irradiation.

Next, we analyzed the impact of conditioning therapy on outcome, and picked a relevant, controversially discussed key difference [29, 30]: when distinguishing between patients who were treated with chemotherapy only and those with TBI-based conditioning, we observed a statistically better survival in the latter group with a 5y-OS of 28.6% vs. 42.4% and a 5y-PFS of 24.2% vs. 40% (both *p* < 0.01; Fig. 3a, b). The cumulative incidence of relapse did not differ (5y-RR 41% without vs. 39.4% with TBI, *p* = 0.751; Fig. 3c). The cumulative incidence of NRM was significantly lower in the TBI-group (5y-NRM 34.8% without vs. 20.6% with TBI, *p* < 0.05; Fig. 3d).

### Patients´ age

**Fig. 4:**
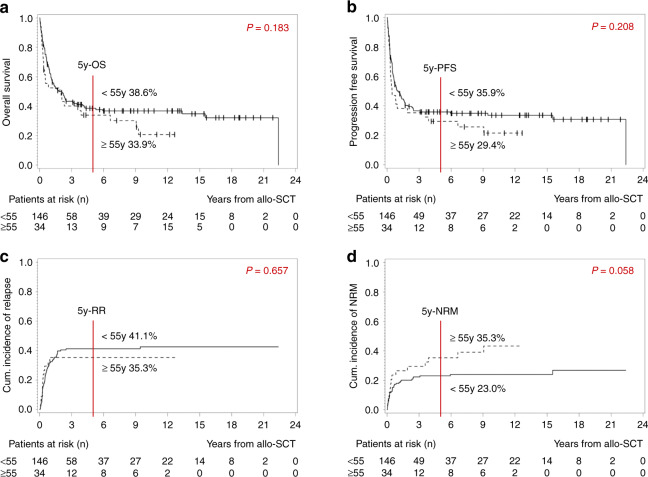
Impact of patients´ age at the timepoint of allo-SCT on outcome: Patients younger than 55 years (*n* = 146) vs. patients 55 years or older (*n* = 34). **a** Kaplan–Meier estimates for OS, **b** Kaplan–Meier estimates for PFS, **c** cumulative incidence of relapse, **d** cumulative incidence of NRM. *OS* overall survival, *PFS* progression free survival, *RR* relapse rate, *NRM* non-relapse-related mortality, *y* year.

As an age limit for allo-SCT is often discussed, a cut-off of 55 years was determined in line with the GMALL-definition for elderly patients to analyze the impact of age on outcome. Notably, survival was similar in both groups (5y-OS 38.6% <55 y vs. 33.9% ≥55 y, *p* = 0.183, Fig. 4a; 5y-PFS 35.9% <55 y vs. 29.4% ≥55 y, *p* = 0.208, Fig. 4b). There was no difference regarding the cumulative incidence of relapse (5y-RR 44.1% <55 y vs. 35.3% ≥55 y, *p* = 0.657; Fig. 4c). The NRM-analysis revealed a trend towards a lower cumulative incidence in younger patients (5y-NRM 23.0% <55 y vs. 35.3% ≥55 y, *p* = 0.058; Fig. 4d).

### Phenotype

Because of the known prognostic relevance of the different immunophenotypes, we compared patients diagnosed with T-ALL, Ph-positive, or -negative B-ALL (Supplementary Fig. 3). There was no difference between those three risk groups regarding OS and PFS (Supplementary Fig. 3A, B) and a trend towards a higher cumulative incidence of relapse in patients diagnosed with T- or Ph-positive B-ALL as compared to Ph-negative B-ALL (Supplementary Fig. 3C). The cumulative incidence of NRM differed significantly between the three phenotypes and was lowest in patients diagnosed with T-ALL (*p* < 0.05; Supplementary Fig. 3D).

### Timepoint of allo-SCT

**Fig. 5:**
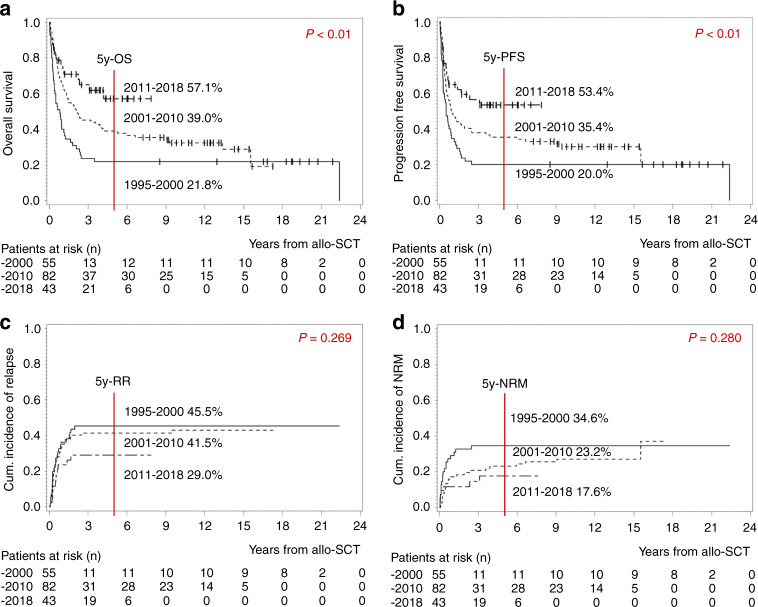
Impact of the timepoint of allo-SCT on outcome: Patients who were transplanted between 1995 and 2000 (*n* = 55) vs. those transplanted between 2001 and 2010 (*n* = 82) vs. those transplanted between 2011 and 2018 (*n* = 43). **a** Kaplan–Meier estimates for OS, **b** Kaplan–Meier estimates for PFS, **c** cumulative incidence of relapse, **d** cumulative incidence of NRM. *OS* overall survival, *PFS* progression free survival, *RR* relapse rate, *NRM* non-relapse-related mortality, *y* year.

When comparing patients transplanted before the year 2000, between 2001 and 2010 and after 2011, we observed a significant improvement over time for both OS and PFS (5y-OS 21.8% vs. 39.0% vs. 57.1%, *p* < 0.01, Fig. 5a; 5y-PFS 20.0% vs. 35.4% vs. 53.4%, *p* < 0.01, Fig. 5b). There was a trend towards a lower cumulative incidence of relapse and NRM for patients in the recent cohort as compared to those transplanted at an earlier timepoint (Fig. 5c, d). Regarding patients´ characteristics, the three cohorts differed significantly as expected (Supplementary Table 2): Due to a lack of therapeutic options, more patients underwent allo-SCT with relapsed/refractory disease in the 1990s (40% vs. 9%; Supplementary Table 2). In earlier years, conditioning therapy was mostly TBI-based. This concept was changed to some degree in the last years with the intention to reduce toxicity (78% vs. 56% TBI-based conditioning; Supplementary Table 2) [30]. In the first cohort, allo-SCT was often performed with BM-stem cells, whereas today, stem cells are almost always harvested from the peripheral blood (42% vs. 2% BM-stem cells; Supplementary Table 2). In the past, more patients received allo-SCT from family members. By contrast, the majority was currently transplanted from unrelated donors (49% vs. 84% unrelated donors; Supplementary Table 2). With this increasing proportion of unrelated donors, the use of antibodies for GvHD-prophylaxis increased over the years (42% vs. 93% Alemtuzumab/Anti-thymocyte Globulin; Supplementary Table 2).

### GvHD

The influence of GvHD on survival after allo-HCT was analyzed in a time-dependent univariate Cox regression model: We observed a significantly impaired survival in patients suffering from aGvHD II° to IV° in comparison to those showing no or aGvHD I° with a Hazard ratio (HR) of 1.47 for PFS (95%-CI 1.02–2.14; *p* = 0.0411) and 1.78 for OS (95%-CI 1.22–2.59; *p* = 0.0028). A similar trend was evident when comparing patients without/with mild cGvHD with those with moderate/severe symptoms (for PFS HR 1.56 [95%-CI 0.96–2.54], *p* = 0.0704; for OS HR 1.45 [95%-CI 0.92–2.28], *p* = 0.1105).

### Multivariate analysis adjusted for remission status at allo-SCT, conditioning regimen, patients´ age, phenotype, and timepoint of allo-SCT

A multivariate Cox regression analysis confirmed the survival benefit for patients who reached MRD-negative CR prior to allo-SCT as compared to those with refractory disease due to an increased RR (*p* < 0.0001; Table 2). Interestingly, there was no difference between cases with MRD-positive or -negative CR and no difference regarding the cumulative incidence of relapse and NRM (Table 2). OS and PFS of patients treated with TBI-based conditioning therapy remained significantly better than survival in the non-TBI group independent of remission, patients´ age, phenotype, and timepoint of allo-SCT (*p* = 0.002; Table 2A). There was no difference concerning NRM and RR (Table 2B). In respect of patients´ age and the three examined phenotypes, the multivariate analysis did not reveal any differences between the corresponding subsets concerning survival, RR, or NRM (Table 2). When comparing survival of patients who received allo-SCT between 2002 and 2010 with those transplanted in recent years, the difference in survival was not statistically significant (Table 2A). The survival advantage between the earliest transplant cohort and the most recent one remained significant (*p* = 0.008 for OS, *p* = 0.033 for PFS; Table 2A) with a trend towards a lower cumulative incidence of NRM (Table 2B).

**Table 2:** Multivariate cox regression analysis for (a) OS and PFS and (b) RR and NRM.

			HR		[95%-CI]		*p* value
(A) OS and PFS							
Remission at allo-SCT	No remission vs. CR MRD neg^a^	OS	3.92		2.54–6.07		<0.0001
		PFS	3.73		2.44–5.70		<0.0001
	CR MRD pos vs. CR MRD neg^a^	OS	1.18		0.70–2.00		0.531
		PFS	1.18		0.71–1.96		0.527
Conditioning	TBI vs. no TBI	OS	0.47		0.29–0.75		0.002
		PFS	0.47		0.29–0.75		0.002
Patients´ age	≥55 vs. < 55 years	OS	0.92		0.49–1.70		0.783
		PFS	0.84		0.46–1.54		0.571
Phenotype	T-ALL vs. B-ALL Ph neg	OS	0.85		0.53–1.37		0.505
		PFS	0.87		0.54–1.38		0.544
	B-ALL Ph pos vs. B-ALL Ph neg	OS	0.75		0.48–1.18		0.213
		PFS	0.71		0.46–1.10		0.128
Timepoint of allo-SCT	1995–2000 vs. 2011–2018	OS	2.28		1.24–4.18		0.008
		PFS	1.89		1.05–3.39		0.033
	2001–2010 vs. 2011–2018	OS	1.62		0.90–2.93		0.111
		PFS	1.50		0.85–2.66		0.163
(B) RR and NRM							
Remission at allo-SCT	No remission vs. CR MRD neg^a^	RR	4.02		2.38–6.80		<0.0001
		NRM	0.95		0.47–1.91		0.881
	CR MRD pos vs. CR MRD neg^a^	RR	1.66		0.82–3.32		0.157
		NRM	0.76		0.36–1.63		0.486
Conditioning	TBI vs. no TBI	RR	0.68		0.36–1.27		0.225
		NRM	0.61		0.29–1.28		0.193
Patients´ age	≥55 vs. < 55 years	RR	0.58		0.24–1.43		0.238
		NRM	1.42		0.59–3.42		0.431
Phenotype	T-ALL vs. B-ALL Ph neg	RR	1.42		0.82–2.46		0.206
		NRM	0.48		0.20–1.18		0.112
	B-ALL Ph pos vs. B-ALL Ph neg	RR	0.55		0.30–1.03		0.061
		NRM	1.32		0.71–2.43		0.377
Timepoint of allo-SCT	1995–2000 vs. 2011–2018	RR	1.41		0.63–3.15		0.400
		NRM	2.56		0.95–6.92		0.063
	2001–2010 vs. 2011–2018	RR	1.91		0.87–4.22		0.108
		NRM	1.40		0.56–3.48		0.468

*HR* Hazard ratio, *CI* confidence interval, *OS* overall survival, *PFS* progression free survival, *CR* complete remission, *MRD* minimal residual disease, *neg* negative, *pos* positive, *TBI* total body irradiation, *Ph* Philadelphia chromosome, *allo-SCT* allogeneic stem cell transplantation, *RR* cumulative incidence of relapse, *NRM* cumulative incidence of non-relapse mortality.

^a^Analysis of Ig-/TR-gene rearrangements via RQ-PCR or surface marker analysis by flow cytometry.

## Discussion

We here analyzed a considerable number of adult ALL-patients who underwent allo-SCT at our center with a long-term follow-up of 10 years. A comprehensive single-center analysis has the advantage of controlled high-quality data and availability of additional details when compared to a registry analysis. By defining specific risk factors derived from subset analyses our data may contribute to the optimization of the allo-SCT procedure in adult ALL.

We observed a significant better survival in patients receiving allo-SCT within their first-line therapy as compared to those with relapsed/refractory disease. However, 64% of the patients transplanted in case of relapse after conventional post-remission chemotherapy (41 of 64) did not achieve CR after salvage therapy prior to allo-SCT, and survival was dismal in this group. In contrast, survival in patients reaching CR2 was almost as favorable as in CR1 confirming the possibility of achieving a second remission with long-term survival [25, 31]. The slightly impaired survival in CR2 may be also caused by an increased NRM due to the advanced disease, cumulative toxicity of previous therapies and patients´ aging [32]. Thus, optimization of re-induction after relapse is crucial, and novel (immuno)therapies with a more favorable side-effect profile should be taken into account as a bridge to allo-SCT in patients who do not achieve CR2 by conventional salvage therapy, such as CAR T-cells [33, 34], inotuzumab [35] and blinatumomab [6, 36]. The importance of a well tolerable and highly efficient induction therapy also became apparent in Ph-positive cases: The outcome of this group has considerably improved due to the additional targeted therapy with TKI such as imatinib [23]. Despite a small cohort of 50 patients and application only since 2006, when it was approved in Europe for this indication, we could confirm a significant survival benefit in the imatinib-cohort. Currently, second- and third-generation TKI are implemented in study protocols, probably leading to an even deeper response [24].

MRD was evaluated in all cases either by analysis of Ig-/TR-gene rearrangements by RQ-PCR [27] or of surface markers by flow cytometry [37, 38]. In a multivariate analysis of our cohort, MRD-status achieved prior to allo-SCT had only a modest impact on survival. In the subgroup of patients in CR1 prior to allo-SCT and with available MRD-assessment via RQ-PCR, the survival benefit in case of MRD-negativity was more distinct but still statistically insignificant. While it has been shown that adult patients with poor MRD-response following induction and consolidation of pediatric-inspired protocols benefit from allo-SCT when compared with continuous conventional chemotherapy [15, 20], it is less clear whether their outcome can be further improved by conversion to MRD-negativity with additional pre-transplant therapy before conditioning. In contrast to our data, some prior studies have found that adult patients reaching MRD-negativity prior to allo-SCT have a superior outcome [39, 40]. However, consistent with our results, there are also studies in pediatric and adult ALL suggesting that MRD-negativity is not an absolute prerequisite for allo-SCT and that MRD post-allo-SCT is more important than pre-allo-SCT [41, 42].

These observations are in line with the view that, probably due to the rapid kinetics of dividing leukemic blasts, an effective GvL-effect after allo-SCT may only be achieved in case of low-level residual disease after conditioning therapy [12]. Accordingly, the influence of conditioning therapy on successful clearance of residual disease and outcome is of interest: Myeloablative conditioning is supposed to confer a survival advantage, as it may lead to a deeper remission with a more effective GvL-effect [11, 43–45]. The optimal conditioning protocol was not yet examined in randomized trials. In line with previous retrospective studies, our data indicate that a TBI-based regimen may be the preferable type of myeloablative therapy [11, 15, 28, 38]: it can induce a more prolonged immunosuppressive effect, prevent distribution variations for example by drug interactions and has an effect on tissues prone to leukemic infestation and with poor chemotherapeutic penetration. Notably, we observed no difference in RR contributing to the difference in survival, but a significantly lower NRM in the TBI-cohort. This difference remained unchanged in the multivariate analysis adjusted for patients´ age, rebutting the presumption that mainly elderly, unfit patients prone to complications were transplanted after chemotherapy-only conditioning in order to avoid a potentially higher toxicity with TBI. However, the impact of the concomitant medication should be taken into account: For example, alemtuzumab was more frequently applied for GvHD-prophylaxis in the non-TBI-group (49% vs. 14%), probably contributing to the increased treatment toxicity in this cohort. Thus, the expected lower toxicity of non-TBI-regimens in our cohort may be outweighed by adverse co-factors.

The experience with allo-SCT in elderly ALL patients is limited, and the potential survival benefit due to a reduced relapse risk may be abrogated by a substantially increased toxicity as compared to younger patients [46]. However, our data suggest that allo-SCT is feasible in carefully selected elderly patients as we did not see a disadvantage in survival in this group. Unsurprisingly, the NRM analysis showed a trend towards a better tolerability in patients younger than 55 years.

In our cohort, survival of patients diagnosed with Ph-negative B-ALL was impaired as compared to Ph-positive B-ALL and T-ALL. We assume an impact of the cumulative toxicity of extensive pre-treatment because this group included more cases who underwent allo-SCT in an individual treatment attempt with lack of alternatives in relapsed/refractory settings (proportion of patients transplanted in CR2 or with relapsed/refractory disease in Ph-negative B-ALL 45% vs. 14% in Ph-positive B-ALL vs. 41% in T-ALL). A meaningful subdivision to prove this presumption was limited by the small sample size of subgroups, but this observation emphasizes the need for optimized re-induction with novel agents.

GvHD is discussed to be associated with an augmented GvL-effect, and thus a lower risk of relapse, but higher NRM may abrogate this favorable effect: data from the International Blood and Marrow Transplant Research-registry suggest that the positive GvL-effect only outweighs the enhanced NRM in case of low-grade aGvHD [12]. Accordingly, we observed an impaired survival in patients exhibiting GvHD of higher grade as compared to those without or with low grade GvHD.

In analogy to comprehensive registry data our analyses confirm a significantly increased survival of ALL-patients who underwent allo-SCT over the last decades [10, 11], due to improved risk stratification and patient selection, adapted treatment protocols for induction/conditioning and reduced NRM as a result of a better supportive therapy and GvHD management.

In conclusion, favorable prognostic factors in our cohort are CR before allo-SCT, TBI as conditioning, and more recent transplantation. There was no difference in outcome in patients older than 55 years, in relation to the MRD-status prior to allo-SCT or between different phenotypes. The findings of our long-term single-center study support that allo-SCT will remain an important therapeutic element in the treatment of adult ALL and help to re-define its role against the background of evolving new therapeutic approaches. To lower NRM, future prospective trials should be designed with combinations of new drugs that induce a deep remission with low toxicity prior to allo-SCT, for example combining immunotherapy with reduced-toxicity conditioning, thus can enhance the efficacy of GvL-effect after transplantation, and lead to a long-term disease control and survival in high-risk ALL-patients.

## Supplementary information

Suppl. tab. 1–2, Suppl. fig. 1–3
