# Correction to: Prognostic factors for survival after allogeneic transplantation in acute lymphoblastic leukemia

**DOI:** 10.1038/s41409-021-01366-y

**Published:** 2021-07-08

**Authors:** C. Greil, M. Engelhardt, G. Ihorst, J. Duque-Afonso, K. Shoumariyeh, H. Bertz, R. Marks, R. Zeiser, J. Duyster, J. Finke, R. Wäsch

**Affiliations:** 1 Department of Hematology, Oncology and Stem Cell Transplantation, Medical Center, University of Freiburg, Freiburg, Germany; 2 Faculty of Medicine, University of Freiburg, Freiburg, Germany; 3 Clinical Trials Unit, Faculty of Medicine, University of Freiburg, Freiburg, Germany

**Keywords:** Acute lymphocytic leukaemia, Risk factors

Correction to: *Bone Marrow Transplantation* 10.1038/s41409-020-01101-z, published online 31 October 2020

The article “Prognostic factors for survival after allogeneic transplantation in acute lymphoblastic leukemia,” written by Greil et al., was originally published electronically on the publisher’s internet portal on October 31, 2020 without open access. With the author’ decision to opt for Open Choice the copyright of the article changed on May 27, 2021 to © Author(s) 2020 and the article is forthwith distributed under a Creative Commons Attribution 4.0 International License, which permits use, sharing, adaptation, distribution, and reproduction in any medium or format, as long as you give appropriate credit to the original author(s) and the source, provide a link to the Creative Commons licence, and indicate if changes were made. The images or other third-party material in this article are included in the article’s Creative Commons licence, unless indicated otherwise in a credit line to the material. If material is not included in the article’s Creative Commons licence and your intended use is not permitted by statutory regulation or exceeds the permitted use, you will need to obtain permission directly from the copyright holder. To view a copy of this licence, visit http://creativecommons.org/licenses/by/4.0. Open access funding enabled and organized by Projekt DEAL.

