# Supplementary material for: Prognostic factors for survival after allogeneic transplantation in acute lymphoblastic leukemia
Source: Bone Marrow Transplant. 2020 Oct 31;56(4):841–52. doi: 10.1038/s41409-020-01101-z (PMC8266681; doi:10.1038/s41409-020-01101-z)
Supplement: Supplementary file 1 — Suppl. tab. 1–2, Suppl. fig. 1–3 [file 41409_2020_1101_MOESM1_ESM.pdf]

Supplementary table 1. Additional information on patients´ characteristics and transplantation procedure (n = 180).

**Body weight** (range)	[kg]	73 (47 - 130)
**Phenotype** (%)	proB- / C- / preB- / mature B-ALL	16 / 83 / 23 / 9 (9 / 46 / 13 / 5)
	early / thymic / mature T-ALL	23 / 13 / 8 (13 / 7 / 4)
	biphenotypic ALL	5 (3)
	tALL	6 (3)
**Therapy prior to allo-SCT** (%)	initial treatment in prospective GMALL trial yes / no	42 / 138 (23 / 77)
	**auto-SCT** yes / no	2 / 178 (1 / 99)
	**TKI** yes / no	29 / 21 (58 / 42) [1]
	**rituximab** yes / no	118 / 62 (66 / 34)
	**WBI** yes / no	86 / 94 (48 / 52)
	**mediastinal irradiation** yes / no	8 / 172 (4 / 96)
**Salvage therapy in case of relapse** (%)	re-induction	20 (31) [2]
	high-dose cytosine arabinoside +/- mitoxantrone	31 (49) [2]
	TKI	4 (6) [2]
	radiation in case of extramedullary recurrence	4 (6) [2]
	blinatumomab	2 (3) [2]
	no / not kown	3 (5) [2]
**Time between ID and allo-SCT** (range) [months]		6.2 (2.0 - 87.7)
**Time between relapse and allo-SCT** (range) [months]		3.6 (0.9 - 30.4)
**Myeloablative conditioning regimen** (%) — TBI	+ etoposide 50mg/kg / + cyclophosphamide 60mg/kg /+ both / + other	47 / 2 / 67 / 3 (39 / 2 / 56 / 3) [3]
no TBI [toxicity-reduced]	fludarabine 3x30 [25] mg/m² + thiotepa 3 [2] x5mg/kg + carmustin 300mg/m² / treosulfan 3x10g/m²	34 [1] / 1 (56 [2] / 1) [4]
	busulfan only 4x3.2mg/kg / + cyclophosphamide 2x60mg/kg / + fludarabine 4x30mg/m²	1 / 9 / 1 (1 / 15 / 1) [4]
	fludarabine 4x30mg/m² + thiotepa 2x5mg/m² / + melphalan 140 [110] mg/m²	4 / 7 [3] (7 / 12 [5]) [4]
	fludarabine 4x30mg/m² + carmustine 2x150mg/m² + melphalan 140 [110] mg/m²	4 [2] (7 [3]) [4]
**Donor sex** (%)	male / female	106 / 74 (59 / 41)
**CMV-status** (%)	donor pos / neg	77 / 103 (43 / 57)
	patient pos / neg	99 / 81 (55 / 45)
**Median engraftment** (range) [day after allo-SCT]	ANC > 500/nl	17 (9 - 32)
	PLT > 20/nl	13 (7 - 150)
**DLI** (%)	yes / no	38 / 142 (21 / 79)
**Time between allo-SCT and DLI** (range) [months]		7.3 (1.0 - 102.4)
**Subsequent allo-SCT** (%)	yes / no	23 / 157 (13 / 87)

Abbreviations: ALL = acute lymphoblastic leukemia; t = therapy-related; GMALL = German ALL study group; auto-SCT = autologous stem cell transplantation; TKI = tyrosine kinase inhibitor; WBI = whole-brain irradiation; ID = initial diagnosis; allo-SCT = allogeneic stem cell transplantation; TBI = total body irradiation; pos = positive; neg = negative; CMV = cytomegalovirus, ANC = absolute neutrophil count; PLT = platelet count; DLI = donor lymphocyte infusion.

[1] as proportion of 50 patients with B-ALL Ph pos; [2] as proportion of 64 relapses; [3] as proportion of 119 patients with TBI; [4] as proportion of 61 patients without TBI.

Supplementary table 2. Patients´ characteristics with regard to the timepoint of allo-SCT.

		1995 - 2000<br>n = 55	2001 - 2010<br>n = 82	2011 - 2018<br>n = 43
**Median age** (range) [years]		33 (16 - 57)	45 (19 - 69)	35 (18 - 79)
**Phenotype** (%)	B-ALL Ph neg	26 (47)	39 (49)	21 (49)
	B-ALL Ph pos	15 (27)	25 (29)	10 (23)
	T-ALL	14 (26)	18 (22)	12 (28)
**Remission state at allo-SCT** (%)	CR1 MRD neg PCR[1] / FACS[2]	7 / 8 (13 / 14)	16 / 21 (19 / 26)	16 / 1 (37 / 2)
	CR1 MRD pos[1+2]	6 (11)	13 (16)	14 (32)
	primary refractory	3 (5)	8 (10)	3 (7)
	CR2 MRD neg PCR[1] / FACS[2]	0 / 7 (0 / 13)	1 / 5 (1 / 6)	3 / 2 (7 / 4)
	CR2 MRD pos[1+2]	2 (4)	3 (4)	0 (0)
	relapsed refractory	22 (40)	15 (18)	4 (9)
**Donor** (%) related / unrelated		28 / 27 (51 / 49)	24 / 58 (29 / 71)	7 / 36 (16 / 84)
**Stem cell source** (%) BM / PB		23 / 32 (42 / 58)	3 / 79 (4 / 96)	1 / 42 (2 / 98)
**TBI-based conditioning** (%) yes / no		43 / 12 (78 / 22)	50 / 32 (61 / 39)	24 / 19 (56 / 44)
**GvHD prophylaxis** (%)	CyA	54 (98)	81 (99)	43 (100)
	+ ATG	23 (42)	23 (28)	34 (79)
	+ Alemtuzumab	0 (0)	41 (50)	6 (14)

Abbreviations: allo-SCT = allogeneic stem cell transplantation; ALL = acute lymphoblastic leukemia;Ph = Philadelphia chromosome; pos = positive; neg = negative; CR = complete remission; MRD = minimal residual disease; TBI = total body irradiation; BM = bone marrow; PB = peripheral blood; GvHD = graft-versus-host disease; CyA = cyclosporin A; ATG = Anti-thymocyte globulin.

[1] analysis of Ig-/TR-gene rearrangements via rtPCR in 11 (20%), 35 (43%) and 39 (91%) patients, respectively;

[2] analysis via surface marker analysis by flow cytometry in 44 (80%), 47 (57%) and 4 (9%) patients, respectively.

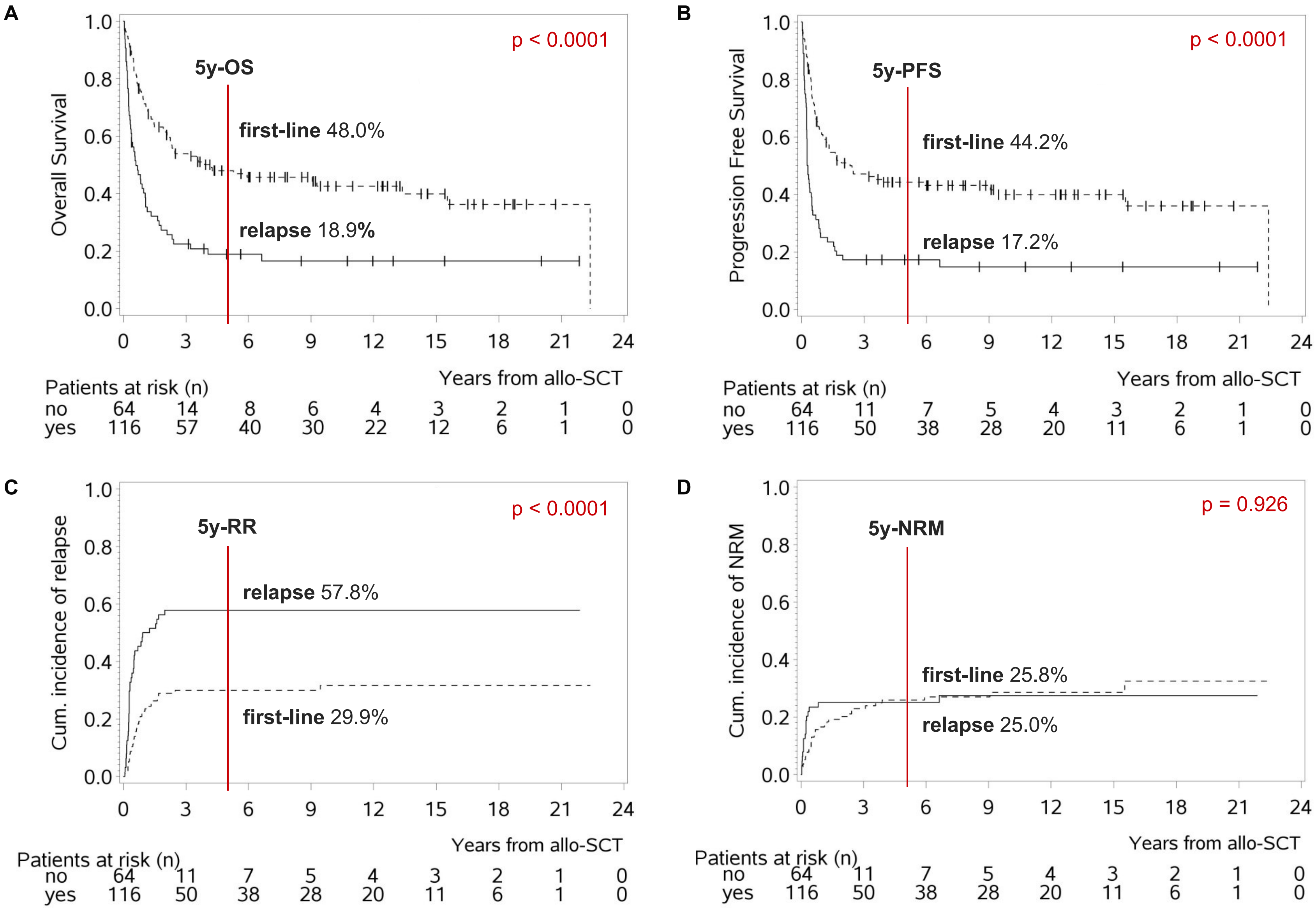

Supplementary figure 1. Impact of therapy line on outcome: Patients who received allo-SCT upfront in the course of their first-line therapy (1. CR and primary refractory; n = 116) vs. thoses transplanted in case of relapsed disease (2. CR and relapsed refractory; n = 64). **(A)** Kaplan-Meier estimates for OS, **(B)** Kaplan-Meier estimates for PFS, **(C)** cumulative incidence of relapse, **(D)** cumulative incidence of NRM.

Abbreviations: allo-SCT = allogeneic transplantation; OS = overall survival; PFS = progression free survival; RR = relapse rate; NRM = non-relapse-related mortality; y = year; CR = complete remission.

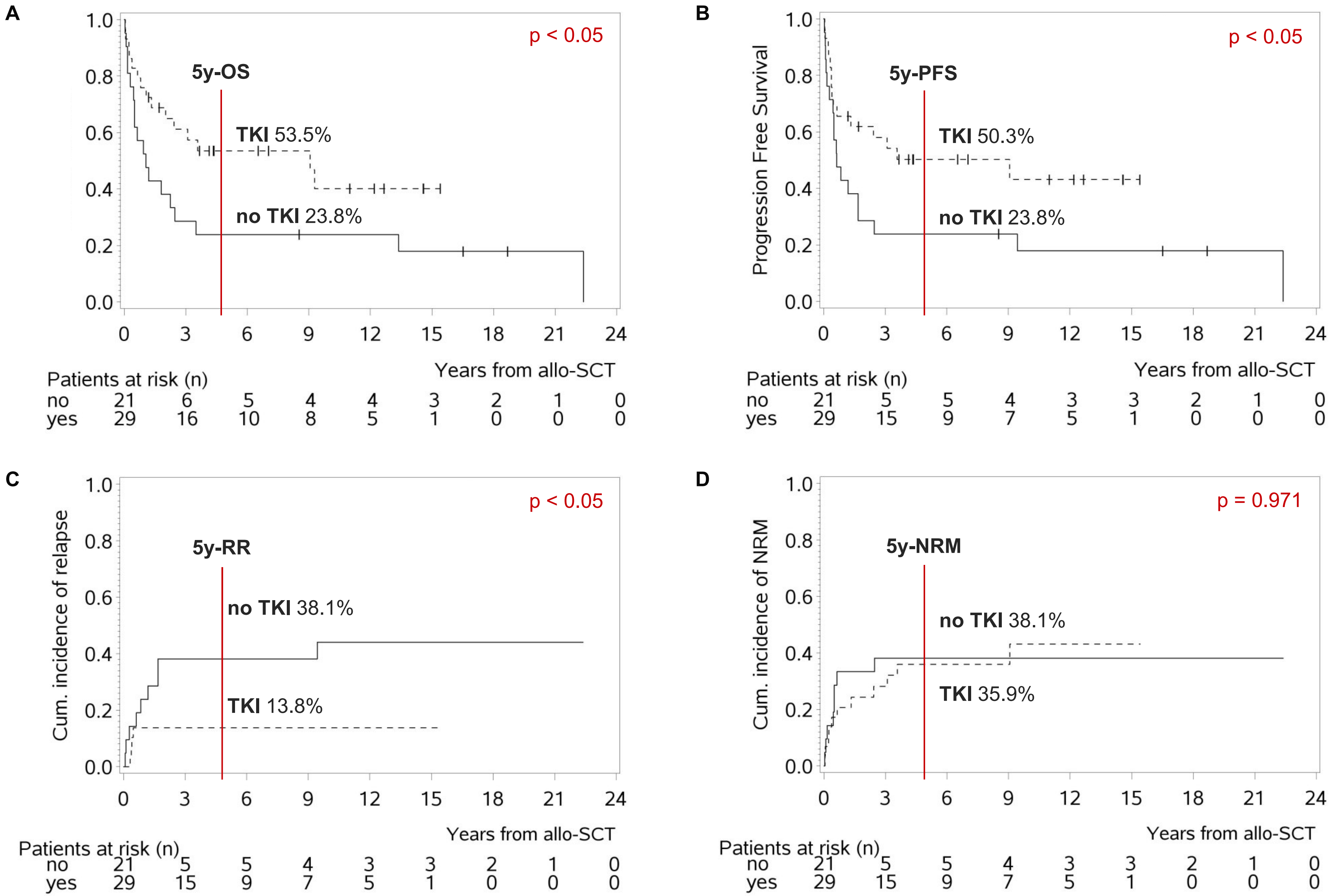

Supplementary figure 2. Differentiation of patients diagnosed with Philadelphia-positive B-ALL (n = 50) treated in the pre-TKI era (n = 21) vs. those who received additional therapy with TKI before allo-SCT (n = 29). **(A)** Kaplan-Meier estimates for OS, **(B)** Kaplan-Meier estimates for PFS, **(C)** cumulative incidence of relapse, **(D)** cumulative incidence of NRM.

Abbreviations: OS = overall survival; PFS = progression free survival; RR = relapse rate; NRM = non-relapse-related mortality; y = year; ALL = acute lymphoblastic leukemia; allo-SCT = allogeneic transplantation; TKI = tyrosine kinase inhibitor.

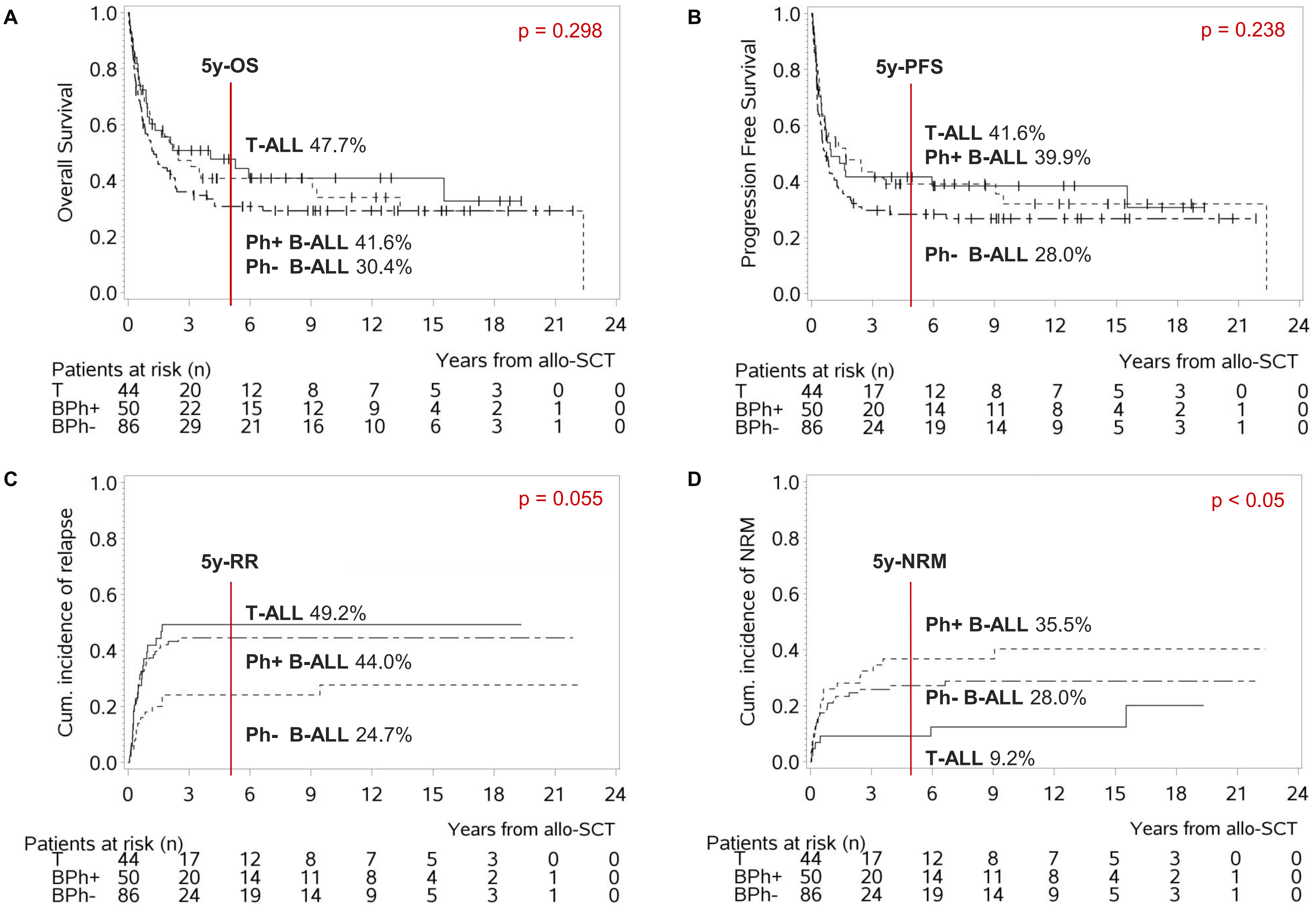

Supplementary figure 3. Impact of phenotype on outcome: Patients diagnosed with T-ALL (n = 44) vs. Philadelphia-positive B-ALL (n = 50) vs. Philadelphia-negative B-ALL (n = 86). **(A)** Kaplan-Meier estimates for OS, **(B)** Kaplan-Meier estimates for PFS, **(C)** cumulative incidence of relapse, **(D)** cumulative incidence of NRM.

Abbreviations: OS = overall survival; PFS = progression free survival; RR = relapse rate; NRM = non-relapse-related mortality; y = year; ALL = acute lymphoblastic leukemia; Ph = Philadelphia chromosome.
